# Copper sulfide nanostructures: synthesis and biological applications

**DOI:** 10.1039/d1ra08414c

**Published:** 2022-03-08

**Authors:** Noor ul Ain, Jamal Abdul Nasir, Zaibunisa Khan, Ian S. Butler, Ziaur Rehman

**Affiliations:** Department of Chemistry, Quaid-i-Azam University Islamabad-45320 Pakistan zrehman@qau.edu.pk hafizqau@yahoo.com +92-(051)90642241 +92-(051)90642245; Department of Chemistry, McGill University 801 Sherbrooke St. West Montreal Quebec Canada H3A 0B8

## Abstract

Over the past few years, considerable attention has been paid to biomedical applications of copper sulfide nanostructures owing to their enhanced physiochemical and pharmacokinetics characteristics in comparison to gold, silver, and carbon nanomaterials. The small-sized Cu_*x*_S_*y*_ nanoparticles have the advantage to absorb efficiently in the near-infrared region (NIR) above 700 nm and the absorption can be tuned by altering their stoichiometries. Moreover, their easy removal through the kidneys overpowers the issue of toxicity caused by many inorganic substances. The low cost and selectivity further add to the advantages of Cu_*x*_S_*y*_ nanostructures as electrode materials in comparison to relatively expensive materials such as silver and gold nanoparticles. This review is mainly focused on the synthesis and biomedical applications of Cu_*x*_S_*y*_ nanostructures. The first part summarizes the various synthetic routes used to produce Cu_*x*_S_*y*_ nanostructures with varying morphologies, while the second part targets the recent progress made in the application of small-sized Cu_*x*_S_*y*_ nanostructures as biosensors, and their analysis and uses in the cure of cancer. Photoacoustic imaging and other cancer treatment applications are discussed. Research on Cu_*x*_S_*y*_ nanostructures will continue to increase over the next few decades, and great opportunities lie ahead for potential biomedical applications of Cu_*x*_S_*y*_ nanostructures.

## Abstract 2

Over the past few years, considerable attention has been paid to biomedical applications of copper sulfide nanostructures owing to their enhanced physiochemical and pharmacokinetics characteristics in comparison to gold, silver, and carbon nanomaterials.

## Graphical abstract

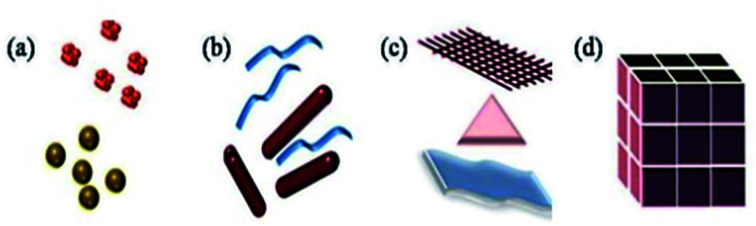

## 1. Introduction

Nanotechnology is gaining much attraction in cancer treatment which can be attributed to its unique features: drug delivery, bioimaging, and the therapeutic nature of nanomaterials. Nanomedicine offers better approaches for diagnosing and treating cancer owing to its enhanced efficacy, reduced toxicity and targeted effect in comparison to chemotherapy, radiation therapy, hyperthermia, immunotherapy and gene or RNA interference (RNAi) therapy that are under clinical investigation.^1–3^ Nanomaterials can be efficiently used as promising materials in many biomedical applications owing to their tunable physiochemical properties.^4,5^ Inorganic nanoparticles, like those of Au, Fe, and Ag, have enhanced optical, ultrasonic, and magnetic characteristics and these have been widely examined for the detection and ultimate cure of tumors *in vivo*. Copper sulfide is gaining considerable popularity in nanotechnology owing to its unique properties that are discussed in this review.^6,7^

Nanomaterials with sizes under 5.5 nm are more favorable for the detection and therapy of cancer as they can be eliminated from the kidneys. The small-sized inorganic nanoparticles (NPs) are expelled quickly without undergoing degradation, in contrast to large-sized nanoparticles. Nanomaterials larger than 5.5 nm (the renal limit) go through the endothelial reticular system and are attached to the lymph, spleen, liver, and other organs, and thus are bio-degraded and then cleared from the body. This procedure prompts a longer stay of NPs in the body bringing about long-lasting toxicity *in vivo* and can lead to many adverse effects.^8–10^ Therefore, the use of small-sized NPs overpowers the issue of toxicity caused by inorganic substances.^11^ The particle size, however, is not the only feature that influences renal clearance.

Other aspects, such as surface characteristics and morphology of the nanostructures can also affect metabolic paths in the human body. NPs smaller than 5.5 nm noticeably increase the area of the exposed surface, leading to modifications in the physical and chemical features of the nanostructures. For instance, the fluorescence quantum yield in the case of 1–2 nm Au clusters is 5 times greater than that for larger Au clusters, while PVP-coated 1.5 nm Au clusters show enhanced catalytic activity. Furthermore, the smaller Au NPs enter the undamaged skin harmlessly and do not cause any structural changes in the skin, thus making it a more suitable option in contrast to transdermal delivery. Cu_*x*_S_*y*_, a p-type semiconductor with outstanding optical characteristics compared to Au and Ag, has been widely used in light emission, charge transport, photocatalysis, and thermal diffusion studies.^4^ Recently, Cu_*x*_S_*y*_ nanostructures have attracted much attention in the biomedical field. In contrast to other NIR absorbing materials, such as Au, Ag, and carbon, Cu_*x*_S_*y*_ materials absorb in the 700–1000 nm range, and the absorptions can be tuned above 900 nm by altering their stoichiometries. Biological tissues exhibit almost no absorptions in this region, which reduces background noise and enhances the spatial resolution of photoacoustic imaging, and also minimizes harm to any healthy tissue. The Cu^2+^ ion (3d^9^) is paramagnetic and can be used as a potential contrast agent. This feature makes Cu_*x*_S_*y*_ nanostructures suitable candidates for multimodal imaging. As already mentioned, in contrast to large-sized CuS nanostructures, smaller-sized ones can be efficiently removed *via* the kidneys making them more biocompatible.^12,13^ The smaller dimensions make it easier to attack the vital parts of a tumor, thus enhancing the possibility of photothermal therapy (PTT). Furthermore, changes in dimensions can likewise produce variations in the properties of the NIR absorption.^14^

Diabetes has harmful effects on body nerves and vessels, which is why careful monitoring is extremely important. Enormous efforts have been undertaken over many years to produce highly sensitive, reliable, and efficient fast response sensors. Previously, enzyme-based glucose biosensors were used, but their activity is mitigated by many factors, such as toxic chemicals, temperature, and pH. Because of this, these sensors now have limited applications. Moreover, the disadvantages diverted the attention of scientists towards non-enzymatic glucose biosensors and ultimately led to the development of the first glucose sensor based on enzyme glucose oxidase about 50 years ago.^15^ Many glucose sensors, based on mesoporous and nanoscale electrodes incorporating metallic and semiconductor nanoparticles have now been produced that allow direct electron transfer between the electrode and the enzyme trapped in the pore. Cu_*x*_S_*y*_ NPs have a metal-like conductivity and provide an alternative to noble metal-based sensors.

DNA sensors are of utmost importance in many laboratory procedures such as gene's analysis, forensic sciences and disease diagnosis, *etc.* Among various techniques discovered for the detection of DNA hybridization, chemiluminescence based on nanomaterials has got much importance. This technique is advantageous over classical chemiluminescence (based on enzymes) owing to enhanced stability and better detection sensitivity. The use of Ag^+^ and Au^3+^ in DNA biosensors are widely reported but they have the disadvantage of poor stability in an aqueous solution. Cu^2+^ based nanomaterials can bypass them owing to their high solubility in water and less cost.^11^

This review describes the recent progress made in the application of small-sized Cu_*x*_S_*y*_ nanostructures as biosensors, and their analysis and their application in the treatment of cancer. Both photoacoustic imaging and some other important cancer treatment applications are discussed.

## 2. Synthetic methods for the production of copper sulfide nanoparticles

**Fig. 1:**
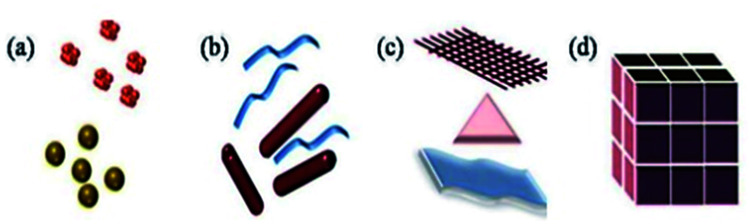
Classification of nanomaterials (a) 0D clusters and spheres (b) 1D nanofibers, rods and wires (c) 2D films, plates and networks (d) 3D nanomaterials. Reprinted with permission from ref. 16, International Organization of Scientific Research, Copyright 2017.^16^

This review article includes a study of a wide range of nanostructures, including 3D nanomaterials, 2D nanoplates and nanofilms, and 1D nanofibers/nanowires (Fig. 1).

Various synthetic routes are available for the generation of nano-sized materials with uniform size, shape, and distribution. These routes make use of chemical methods for the fabrication of the materials and inhibit particle growth by controlling the reaction conditions. This approach is advantageous for industrial applications as it is important to regulate grain size, crystalline phase, morphology, and surface chemistry through the control of the solution composition, applied pressure, reaction temperature, and presence of any additives, solvent properties, and aging time.

Various analytical techniques, including X-ray diffraction (XRD), scanning electron microscopy (SEM), Fourier transform infrared spectroscopy (FTIR), energy dispersive X-ray spectroscopy (EDS), atomic force microscopy (AFM), transmission electron microscopy (TEM), high-resolution TEM (HRTEM), dynamic light scattering (DLS), and UV-visible and photoluminescence (PL) spectroscopy can be used in the characterization of Cu_*x*_S_*y*_ NPs. These techniques provide useful information regarding the structural, elemental, and optical properties of Cu_*x*_S_*y*_ NPs.

### 2.1 Copper sulfide nanospheres and nanocages

Copper sulfide nanospheres have been successfully synthesized *via* hydrothermal, solvothermal, combustion, microwave, gas phase, sol–gel, and sonochemical methods.^17–20^ Although each of these processes has its own advantages, the hydrothermal method is the preferred one owing to the ready availability of water, the low cost, and the environmentally friendly nature. This method makes use of water because of its ability to dissolve nonionic covalent compounds under high temperature and pressure conditions. Water acts as both a pressure-transmitting medium and a solvent medium. These properties of water act collectively to lower the activation energy of the reactions. For example, Cu (CuO, CuCl_2_·2H_2_O, *etc.*) and S (Na_2_S, Na_2_S_2_O_3_ 5H_2_O, thiourea, *etc.*) precursors can be heated in an autoclave at 130–170 °C for a limited number of hours to produce Cu_*x*_S_*y*_ NPs of ∼13 nm in diameter.^17,19^ In another study, ∼10 nm-sized Cu_*x*_S_*y*_ NPs have been created by mixing sodium citrate with aqueous solutions of CuCl_2_ and Na_2_S at ambient temperature followed by heating at 90 °C for about 15 min.^21^ The resulting dimensions and morphologies of the NPs can be modified in this hydrothermal process by changing the precursors, reaction temperature and reaction time.

Among recently established approaches to prepare Cu_*x*_S_*y*_ NPs, microwave irradiation shows great promise owing to its simplicity and energy efficiency.^22,23^ Using CuCl_2_ and Na_2_S as the starting materials, microwave irradiation (180 W) in ethylene glycol or an aqueous medium for about 20 min leads to the formation of Cu_*x*_S_*y*_ NPs. Other, less commonly reported methods to fabricate Cu_*x*_S_*y*_ NPs make use of carboxylic acids as the reaction medium for an enhanced nucleation rate resulting in stabilizing NP suspensions and the surfactant-based creation of Cu_*x*_S_*y*_ NPs.^24^ Empty nanospheres and nanocages have attracted a great deal of attention owing to their chemical storage capacities for use in drug delivery and catalysis. Another report on the formation of empty copper sulfide nanostructures was dependent on the self-assembly of nanoflakes resulting from the use of a Cu^2+^–thiourea compound and NPs of varying diameter were produced.^25^ In addition, both empty Cu_*x*_S_*y*_ nanospheres and nanotubes (5–10 nm diameter) at ambient temperature have been produced.^26^ It is possible that these empty nanostructures are being formed because of the decomposition of thiourea generating H_2_S, which then reacts with the copper precursor to yield CO_2_ gas. The presence of CO_2_ creates gaseous voids, which under hydrothermal conditions work as heterogeneous nucleation centers for the formation of copper sulfide nanoflakes, eventually leading to empty nanostructures.

A hard-template-assisted method has also been employed to produce copper sulfide NPs with enormous empty cavities.^27^ For this technique, distinct types of fundamental supports have been utilized, comprising surfactant micelle micro emulsions,^28^ Cu_2_O nanoparticles,^11,13^ and poly-(styrene-acrylic) latex particles.^29^ Cubic-star-shaped and octahedral NPs, have been used as sacrificial prototypes to create empty Cu_*x*_S_*y*_ nanostructures with sizes ranging from 500 nm to even microns.^30,31^ During this procedure, the prototypes are encompassed in a suitable solvent resulting in the formation of a sulfide shell around them. Such template-based development may be either due to the Kirkendall diffusion effect or the process of mass diffusion followed by Ostwald ripening.

### 2.2 Copper sulfide nanoplates

Apart from the 3D nanomaterials mentioned above, 2D CuS/Cu_2_S nanostructures are comparatively uncommon and are mostly limited to nanoplates and thin films.^32,33^ The most common synthetic methods for the synthesis of CuS/Cu_2_S nanoplates involve hydrothermal and solvothermal approaches that yield nanoplates with the size range of 50–200 nm depending on the experimental conditions employed.^19,34–37^ Some research has been reported involving a surfactant-based aqueous micro-emulsion method at 130–180 °C for a few hours.^38,39^

The sonochemical method is also useful for the preparation of nanoplates. This approach can be advantageous in the preparation of nanoparticles, nanospheres, nanotubes, and nanowires.^40^ By employing the sonochemical method, 20–40 nm-sized nanoplates were synthesized with high crystallinity under standard conditions using Cu(OH)_2_ nanoribbon templates.^40^ Other synthetic processes for Cu_*x*_S_*y*_ nanoplates involve the so-called single-source method,^41^ chemical vapor reactions,^37^ and a high-temperature precursor injection method.^24^

Relatively rare 2D Cu_*x*_S_*y*_ nanoplates can also be obtained *via* the hydrothermal method. Nanoplates with a thickness of 15 nm and an edge length of 60 nm were obtained by treating the cation surfactant cetyltrimethylammonium bromide (CTAB) with CuCl_2_·2H_2_O and Na_2_S·9H_2_O at 180 °C for about 24 h. Hexagonal nanoplates were formed.^36^

### 2.3 Copper sulfide nanotubes, nanorods, and nanowires

Nowadays, 1D nanostructures, such as Cu_*x*_S_*y*_, are extensively studied owing to their detection and photocatalytic applications.^42,43^ These extended nanostructures are especially employed as sensors because of their enhanced catalytic and electrochemical characteristics.

Numerous methods have been investigated to generate these 1D Cu_*x*_S_*y*_ nanomaterials; the process parameters used greatly affect the form/size of the products. For instance, sizes range for nanowires from 30–80 nm (ref. 44 and 45) and 30–120 nm for nanotubes,^18,46^ depending on the composition, growth conditions, and precursors involved. Most methodologies incorporate hydrothermal processes that afford an important approach to create high quality, uniform nanostructures with enhanced precision.^18,26,29,44,46,47^ In the meantime, the thermolytic decrease of a copper thiolate precursor,^48^ the utilization of a paired cell at room temperature,^49^ and a synthesis directed by amylose^50^ have additionally been explored for the synthesis of these copper sulfide NPs. Numerous researchers have synthesized nanorods and Cu_*x*_S_*y*_ tubular structures using a microwave irradiation method under strict conditions. Other methods have been reported that include template-assisted or surfactant-based routes.^51–54^ In a recent study, the manufacture of Cu_*x*_S_*y*_ nanorods exhibiting arachidic acid monolayers was achieved on graphite containing inserted Cu ions.^53^ This technique produced a manageable combination of nanowire arrays on a broad range of amphiphilic Langmuir–Blodgett films that exhibited the required characteristics for possible use in sensors. Another new report has presented data on a microwave-induced heating method for the synthesis of copper sulfide nanorods. The XRD patterns revealed highly pure Cu_*x*_S_*y*_, consisting of uniform and evenly distributed rods with lengths ranging from 30–50 nm and diameters from 5–10 nm. The pure product was highly crystalline with a uniform morphology.^45^ Thongtem *et al.* presented the synthesis of flower-like tubular structures that depended on the pH and irradiation time. This technique made use of CuCl_2_·2H_2_O and CH_3_CSNH_2_ mixed in ethylene glycol along with some NaOH addition to yield solutions of different pHs. These reactions were performed in the absence of surfactants in an acid digestion bomb employing microwave radiation.^55^

## 3. Biomedical applications of copper sulfide NPs

**Fig. 2:**
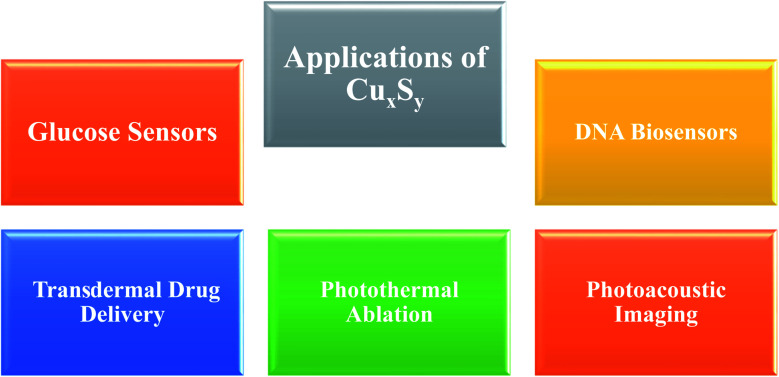
Biomedical applications of Cu_*x*_S_*y*_ nanoparticles.

Copper sulfide NPs have found a variety of biomedical applications, including photoacoustic imaging, photothermal agent, transdermal drug delivery systems, glucose sensors, and DNA sensors (Fig. 2).

### 3.1 Copper sulfide applications in cancer

#### 3.1.1 Copper sulfide in photoacoustic imaging

**Fig. 3:**
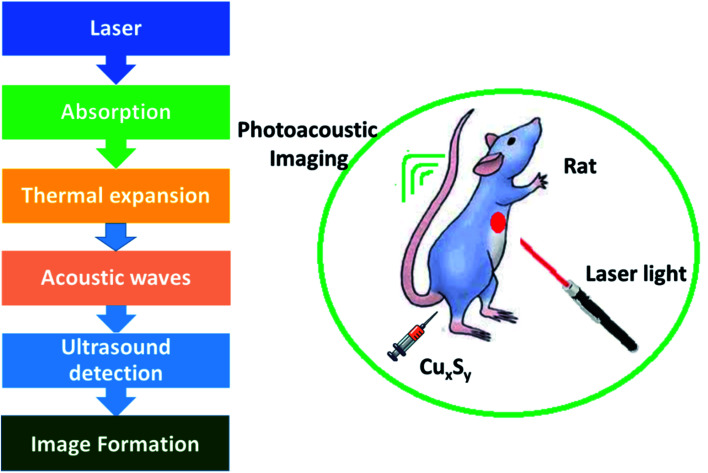
Steps involved in photoacoustic imaging.

Photoacoustic imaging (PAI) is a novel, non-invasive biomedical imaging technique that has been established recently. Photoacoustic signals are produced by any *in vivo* molecule, such as DNA/RNA, hemoglobin, water, lipids, cytochromes, and melanin or Au and CuS NPs (external contrast agents), which absorb short-pulsed laser irradiation for photothermal conversion.^56–58^ In contrast to other advanced optical imaging techniques, PAI involves using low-frequency ultrasounds to generate the required biosignals. The advantage of this technique is that one can imagine a few inches deep inside biological tissue. This method can be used to monitor brain structure and function to detect solid tumors and in tumor angiogenesis.^59–61^Fig. 3 shows the steps involved during photoacoustic imaging.

Photoacoustic imaging has problems in the visible and NIR-I regions, as the radiation can be absorbed and dispersed by organic biological molecules thus impairing the quality of the imaging. Photons in the second region of NIR (1000–1700 nm) are less absorbed and dispersed by biological tissues. Consequently, choosing a material with optical absorption in NIR-II region should efficiently increase PAI quality. Expensive metals and carbon-based NPs, *e.g.*, gold nanocages, nanorods and nanoshells, hollow gold nanospheres (HAuNS), carbon nanotubes, and graphene, display enhanced efficiency of photothermal conversion with improved light stability, and these materials have been explored as possible PA contrast agents. The maximum absorption lies somewhere in the range of 500–800 nm and the materials mentioned exhibit much less absorption in the NIR-II region in comparison to copper sulfide NPs.^62–64^

**Fig. 4:**
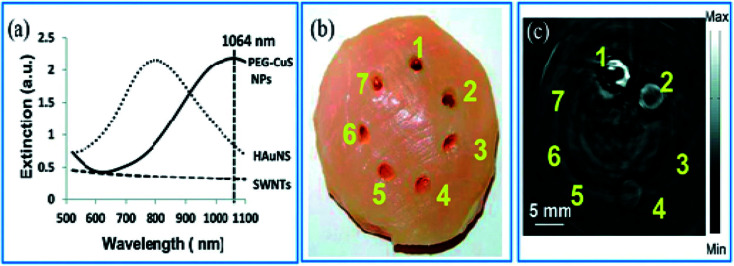
(a) Optical absorption of CuS NPs, HAuNS, and SWNTs at the concentration of 100 μg mL^−1^. (b) Picture and (c) corresponding PAT image of nanoparticles embedded in a chicken thoracic muscle, which was then put under a chicken breast muscle. PAT image was obtained by laser irradiation. 1–3 are 100 mg mL^−1^ of CuS NPs, HAuNS, and SWNTs, respectively. 4–6 are 20 mg mL^−1^ of CuS NPs, HAuNS, and SWNTs, respectively, and 7 is gel without contrast agent. Reprinted with permission from ref. 65, Royal Society of Chemistry, Copyright 2014.^65^

Zhou *et al.*^65^ have evaluated the optical properties of single-walled carbon nanotubes (SWNTs), HAuNSs, and polyethylene glycol-coated CuS NPs (PEG-CuS NPs), under 1064 nm pulsed laser irradiation. Agarose gel, which contains PEG-CuS NPs, SWNTs, and HAuNSs, was inserted under a 1 cm thick chicken breast tissue to obtain a photoacoustic image. Their research revealed that the gel consisting of PEG-CuS NPs yielded a useful signal for a 100 μg mL^−1^ concentration, while the signal strength of gel comprising HAuNSs was comparatively weak, and the SWNTs exhibited no signal at all. Moreover, the PEG-CuS NPs signal was only just detectable for a 20 μg mL^−1^ concentration (Fig. 4).

The NIR absorption of Cu_2−*x*_S NPs involves a d–d transition from Cu^2+^ ion and is almost unaffected by their form and structure. Moreover, the low cost and less toxicity of Cu_2−*x*_S NPs are advantageous for their use in photoacoustic imaging applications.^66^ Jianlin and colleagues have synthesized monodisperse Cu_2−*x*_S nanostructures with enhanced light absorption and conversion efficiency that work in the 600–1100 nm range in comparison to Au nanoparticles. The Cu_2−*x*_S NPs are expelled *via* feces in five days. A 3 month *in vivo* toxicity study showed that the Cu_2−*x*_S NPs had insignificant side effects on blood and tissue and could be considered bio-secure.^66^ The ultrasound contrast agent PFP (perfluoropentane) and Cu_2−*x*_S NPs were loaded by the researchers into the liposomes. At this point, the Cu_2−*x*_S NPs absorbed near-IR radiation producing enough thermal heat for photoacoustic imaging. Increasing the temperature led to a CO_2_-enhanced ultrasound for attaining NIR thermal/PA/ultrasonic trimodal imaging.^67,68^

Liang *et al.* have utilized a modified glutathione (GSH) for developing GSH-Cu_2−*x*_S NPs. The contrast intensity of the photoacoustic signal depends on the concentration of the NPs. A photoacoustic signal still can be produced by a GSH-CuS aq. dispersion having the concentration of copper as low as 1 mM. Notably, the small size and zwitterionic glutathione coating permits the Cu_2−*x*_S NPs to be removed *via* kidneys. Liang *et al.* studied the renal clearance by analysing the urinary excretions, and examined the bio distribution of the synthesized materials in various organs.^68^

**Fig. 5:**
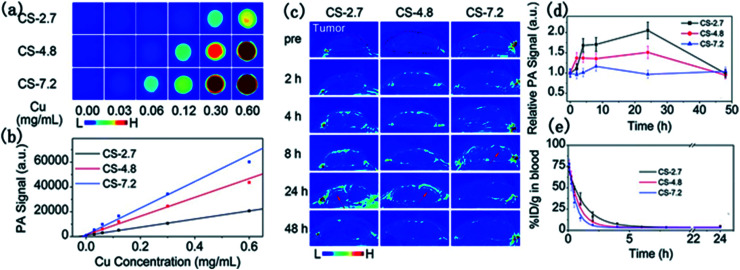
*In vitro* PAI (a) and the corresponding quantified PA signal (b) of aqueous solutions containing differently sized PEG-Cu_2−*x*_S NPs. (c) Photoacoustic images of mouse tumor sites at different times after injection of different sizes of PEG-Cu_2−*x*_S NPs. (d) Blood clearance curves of different sizes of PEG-Cu_2−*x*_S NPs. (e) Theoretical fit curves obtained by a single-chamber model for depicting the removal behavior of PEG-Cu_2−*x*_S NPs. Reprinted with permission from ref. 69, Royal Society of Chemistry, Copyright 2015.^69^

To examine the size effect on photoacoustic imaging, Ding *et al.*^69^ have synthesized PEGylated Cu_2−*x*_S NPs with 2.7, 4.8, and 7.2 nm sizes by governing the reaction time. These researchers inspected the influence of these Cu_2−*x*_S nanostructures on the enhancement of PAI in both *in vivo* and *in vitro* situations (Fig. 5).

There was a positive correlation of NP sizes with the PAI contrast enhancement, and the 7.2 nm PEG-Cu_2−*x*_S nanostructures delivered the best photoacoustic signal. *In vivo* studies demonstrated better tumor imaging performance by the 2.7 and 4.8 nm sized Cu_2−*x*_S nanoparticles in comparison to the 7.2 nm Cu_2−*x*_S nanostructures. The small-sized NPs enter deeper inside the tumor and showed faster blood clearance. Ultrasonography establishes the Cu_2−*x*_S NPs as potential photoacoustic imaging agents (Fig. 5).

Santiesteban and coworkers have managed to synthesize copper sulfide perfluorocarbon nanodroplets (CuS-PFCnDs) with improved acoustic and optical properties that would be suitable for photoacoustic/ultrasound imaging. The PA/US characteristics of CuS-PFCnDs were examined in tissue-mimicking models. A 1 × 10^6^ nDs per mL concentrated solution of CuS-PFCnD was inserted in a gel phantom. The sample and phantom were irradiated with 1064 nm laser light to obtain photoacoustic/ultrasound responses of CuS-PFCnDs. 6–9 mJ cm^−2^ fluences were targeted nearly 1 cm under the transducer and found sufficient to trigger the CuS-PFCnDs response. Upon irradiation, most of the PFCnDs evaporated leading to a rapid and intense photoacoustic signal. There is an apparent decay of the photoacoustic signal over time. Ultrasound contrast shows the opposite response, increasing quickly with the transformation of nanodroplets into microbubbles. With the increase in laser shots, the area becomes filled with microbubbles and the US contrast reached to its maximum. The distinctive features of the photoacoustic signal and ultrasound of PFCnDs can be used to obtain the best PA/US images *in vivo*.^70^

#### 3.1.2 Copper sulfide in photothermal therapy

Photothermal therapy is a new, non-invasive, and operative therapy that makes use of materials which absorb NIR light for producing heat upon laser irradiation. This approach leads to a rise in temperature of the local tumor with irreversible injury to its cells. The lower cost and negligible side effects of photothermal therapy are great benefits when compared to the old cancer treatments such as surgery, radiation therapy, and chemotherapy.^71,72^

**Fig. 6:**
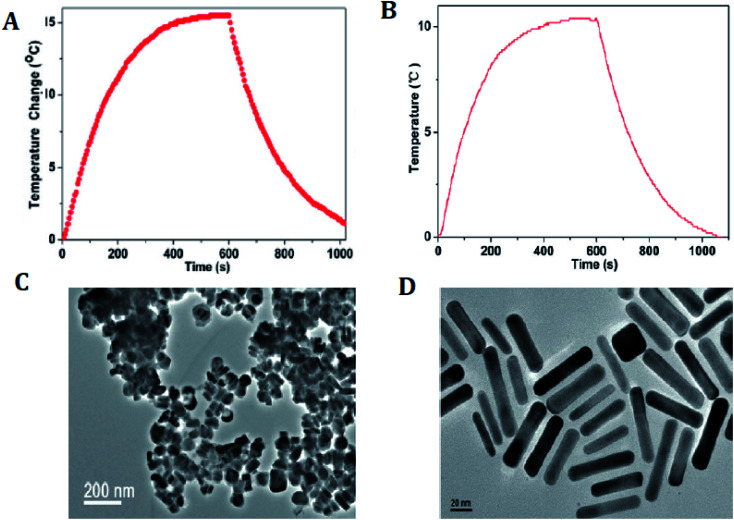
(A) Photothermal effect of the aqueous dispersion of Cu_9_S_5_ NCs (40 ppm) with the NIR (980 nm, 0.51 W cm^−2^) irradiation for 10 min. (B) Photothermal effect of the aqueous dispersion of the Au nanorods (40 ppm) irradiated with 980 laser (a power density of 0.51 W cm^−2^) for 10 min. (C) TEM image of the Cu_9_S_5_ NCs. (D) TEM image of the Au nanorods. Reprinted with permission from ref. 73, American Chemical Society, Copyright 2011.^73^

Numerous Cu_*x*_S_*y*_ nanostructures have been reported as efficient transducers in photothermal ablation therapy, especially flower-like structures^74^ with an average size of ∼1 μm and plate-like nanocrystals^73^ with dimensions of ∼70 nm × 13 nm. These NPs possess strong absorption upon irradiation with a 980 nm laser and exhibit enhanced conversion efficiency resulting in an increase in temperature that helps to destroy the cancer cells (Fig. 6).^73^

**Fig. 7:**
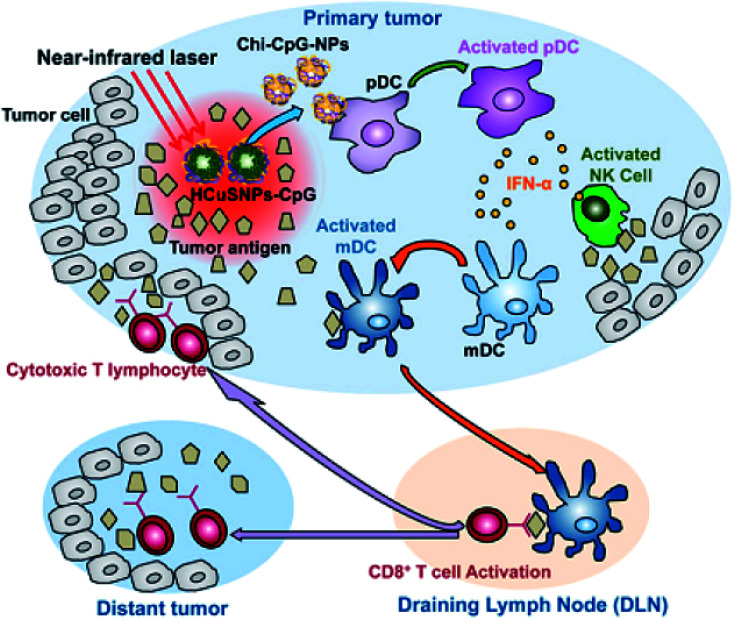
Diagram of HCuSNPs–CpG-mediated photothermal immunotherapy for the treatments of primary and distant tumors. Reprinted with permission from ref. 75, American Chemical Society, Copyright 2014.^75^

Hollow Cu_*x*_S_*y*_ nanostructures with pores afford the possibility of performing a combined photothermal and chemothermal therapy. These materials can efficiently load drugs, *e.g.*, 130 nm diameter hollow structures can be loaded with the hydrophobic drug camptothecin.^73^ The release of this drug in a chemical toxicity study involving hollow Cu_*x*_S_*y*_ nanostructures and NIR light irradiation generated heat from the copper sulfide material and resulted in cell death. Copper sulfide NPs can also be used for combinational photothermal-immunotherapy. For this approach, chitosan-coated hollow copper sulfide nanostructures were combined with CpG motifs (immunoadjuvants) to form a nanocomplex. Following NIR irradiation, death of the tumor cells by photothermal ablation releases tumor antigens into the surrounding environment leads to the reassembly of a chitosan–CpG complex with enhanced immune response. The results indicate that this combined therapy can be used to treat both primary tumor and distant metastasis tumors (Fig. 7).

**Fig. 8:**
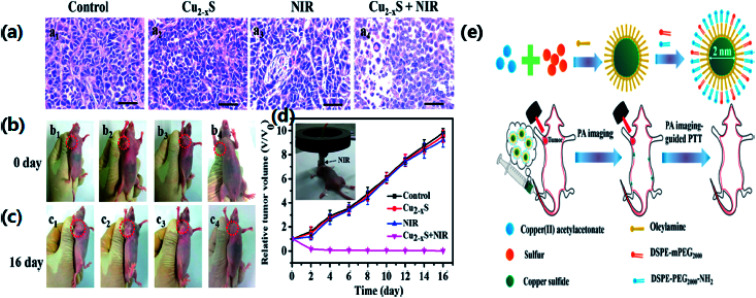
(a) H&E stain of tumor sections of mice in different treatment groups. (b, c) Photographs at 1 and 16 days of mice from different treatment groups. (b1, c1) Saline; (b2, c2) u-Cu_2−*x*_S NDs only; (b3, c3) laser only; (b4, c4) treated with s-Cu_2−*x*_S NDs and laser. (d) Tumor growth curves of different treatment groups. (e) The schematic diagram for the synthetic process of u-Cu_2−*x*_S NDs. Reprinted with permission from ref. 66, Wiley VCH, Copyright 2015.^66^

Owing to the NIR absorption properties of copper sulfide, Li *et al.*^76^ have synthesized copper sulfide NPs for photothermal ablation therapy by a wet chemical method with an average size of 3 nm. They investigated the absorption of light and the photothermal conversion efficiency by irradiating with a NIR laser (∼808 nm). The experimental results showed an increase in temperature of the aqueous solution of Cu_2−*x*_S nanostructures with an increase in contact time and the NP concentration. These NPs exhibited an improved photothermal cell-destroying ability. The cytotoxicity of the copper sulfide nanostructures was compared with that of 20 nm Au nanostructures, which are broadly acknowledged as biocompatible nanoparticles. The copper sulfide NPs can be considered as possible photothermal agents for the first time. PTT monitoring could identify correctly the site, form and size of a tumor, thus avoiding needless injury to healthy tissues. Tracking the biodistribution of such photothermal agents would lead to an efficient and benign therapy. Photothermal conversion could be performed by both PAI contrast and photothermal agents. Thus, the monitoring of photothermal agents for therapies could be achieved using a photosensitizer. Mou *et al.*^66^ have examined oleylamine as a blocking agent for synthesizing small NPs with a biomimetic phospholipid as a surfactant. A clear heating effect was observed upon irradiation with a 980 nm laser, while enhancement of the photoacoustic signal occurred upon irradiation with a 1064 nm pulsed laser. After identification by PAI, the tumor was irradiated with a NIR laser. The results showed a 10 °C increase in the temperature of tumor center. The tumor completely vanished in 2 days when compared with a non-injected NP group, and there was no reappearance detected within 16 days indicating the enhancement in the cell-destroying ability of Cu_2−*x*_S nanostructures under the combined effect of PTT and PAI (Fig. 8). Most of the NPs were expelled through the liver owing to a substantial rise in the size of the particles (∼12 nm).

A multifunctional nuclear/satellite nanostructure with silica-coated rare-earth UCNPs (NaYbF_4_:2% Er^3+^/20% Gd^3+^@SiO_2_–NH_2_) as the core and small CuS NPs as satellites have been developed by Xiao *et al.*^77^ These NPs were used to thermally ablate a tumor while monitoring by MR-CT-optical imaging. Wang *et al.*^78^ have synthesized 5 nm-sized NPs by trapping intermediates. In comparison to 30 nm nanostructures, these small-sized NPs exhibited a wider absorption profile with the maximum absorption at 1200 nm. This difference might have been triggered by their diverse crystallinity and frailer light absorption owing to restrictions caused by their quantum size. The particle size, form, and surrounding medium hardly affect the absorption wavelength, while the intensity of absorption depends greatly on the particle dimensions. Consequently, copper sulfide nanostructures usually need high-power lasers that limit their application for any photothermal work. Several scientists have examined ways to enhance the photothermal ratio of larger-sized nanomaterials. Briefly, they have presented three tactics to enhance the physicochemical characteristics of copper sulfide nanostructures and their photothermal ratios. The first approach involves synthesizing NPs with varying structures to improve their photon refraction properties. The second approach includes the preparation of nonstoichiometric copper sulfide with adjusted copper deficiencies to improve the LSPR (Localized Surface Plasmon Resonance) absorption. The last approach involves synthesizing hybrid composites for enhancing the LSPR absorption properties by coupling the effects of various functional groups. Li *et al.*^76^ have suggested the possibility of obtaining stronger absorption strengths for Cu_2−*x*_S NPs on the basis of theoretical calculations. A practical approach might be to passivate a zinc sulfide (ZnS) surface to restrict the movement of excitons to the core. A surfactant with a compact structure can be applied to formulate nanoparticles with comparatively large core sizes and the least possible hydrodynamic dimensions to concurrently improve the absorption of light and the subsequent kidney metabolism. The addition of ligands with an appropriate surface could also be a feasible choice. These ligands would enhance biocompatibility by layering the exterior of the copper sulfide nanostructures with decomposable substances. Surface ligands might also lead to improvements in the overall characteristics of NPs together with the associated metabolism effects. Many unsaturated bonds result in the formation of several defects on the surface of NPs, which can react with other atoms and alter the characteristics of the whole nanoparticles. This situation occurs because a decrease in nanocrystallites will increase the number of surface atoms. For instance, coating the surface of a CdSe quantum dot with a ZnS shell could alter its light absorption ability and increase the quantum yield. The choice of suitable surface ligands is troublesome as they have a complex influence on the digestion of copper sulfide nanostructures. Studies on the renal clearance of nanoparticles have shown that inorganic metal NPs with sizes less than 5.5 nm can be swiftly eliminated through the kidneys.^79^ Nevertheless, even though the exact principle is unclear, this research has established that structure and chain length do affect the size and metabolism of copper sulfide nanostructures.

#### 3.1.3 Copper sulfide in transdermal drug delivery systems

**Fig. 9:**
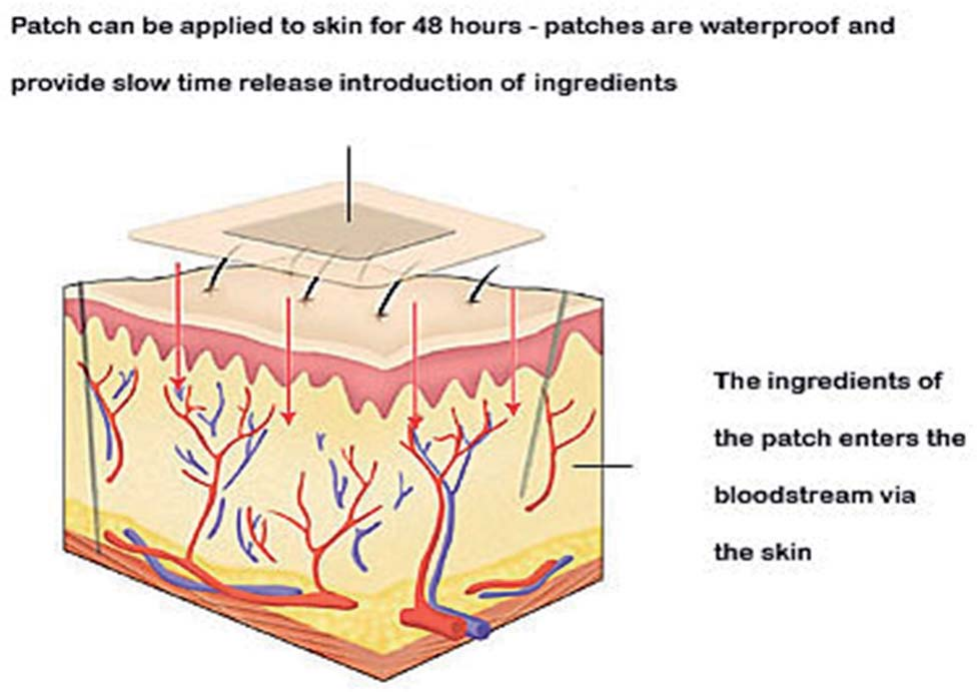
Transdermal drug delivery system.

Cancer chemo- and radiotherapy have many shortcomings, such as drug short half-lives and toxicity problems. These side effects have led scientists to develop new strategies for cancer therapy. Transdermal drug delivery (Fig. 9) is an interesting alternative way to deliver active ingredients. This approach makes use of transdermal patches on the skin and is favored over injections.^80–82^

Skin is a natural body defense that provides protection against foreign substances. The outmost layer is the stratum corneum (SC), which consists of dead keratinocytes together with lipids.^83^ This layer is 10–20 μm thick and it only allows the lipophilic drugs with molecular weight (<500 daltons) to pass through. Much research has been undertaken to enhance the permeability of this skin layer. Chemical enhancers, such as azone (1-dodecylazacycloheptan-2-one) and peptides, have been used to disrupt the lipid bilayer structure of the SC. Some physical methods to disrupt the SC structure involve mechanical and thermal approaches. These disruptions create channels that allow the drug macromolecules to pass through the layer.

**Fig. 10:**
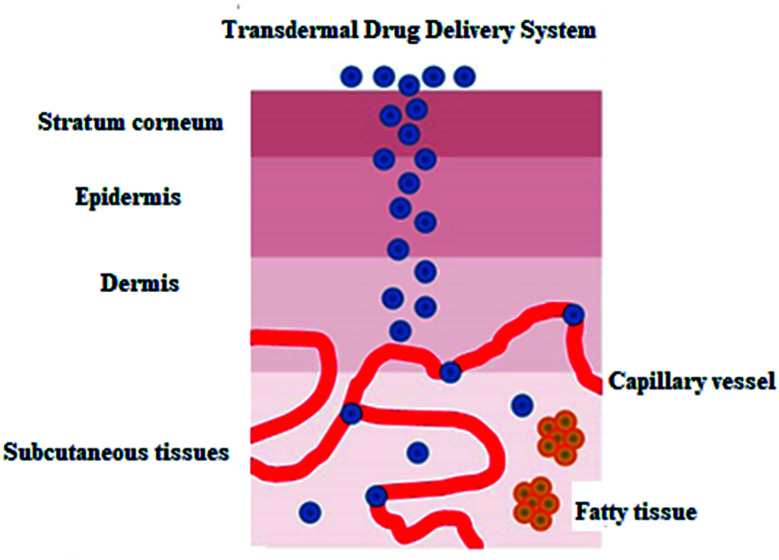
Figure showing the route of transdermal drug delivery. Reprinted with permission from ref. 84, Dovepress, Copyright 2015.^84^

Thermal ablation is most often used for disrupting the stratum corneum (Fig. 10). It employs heat sources like micro heaters,^83^ radio frequency,^85–87^ superheated steam ejected,^88^ or lasers.^74,89–92^ Laser light energy is absorbed by the water and pigments present, which is converted into heat leading to skin thermolysis. The duration of heat is kept in microseconds to avoid penetration to deeper tissues.

Copper sulfide NPs are a useful alternative to Au NPs for photothermal work.^93–96^ The optical absorption in Au NPs originates from a surface plasmon resonance (SPR),^97^ whereas that in CuS NPs originates from a d–d electronic transition. The SPR peak is dependent on the dielectric constant and so a change in the medium will have an effect on it, but this is not the case for CuS NPs. Irradiation of CuS NPs with NIR light under both *in vivo* and *in vitro* conditions leads to the thermal destruction of tumor cells. Nanosecond-pulsed NIR lasers have the advantage providing very high temperatures in a local region for very short period of time, thus protecting the deeper healthy tissue.

**Fig. 11:**
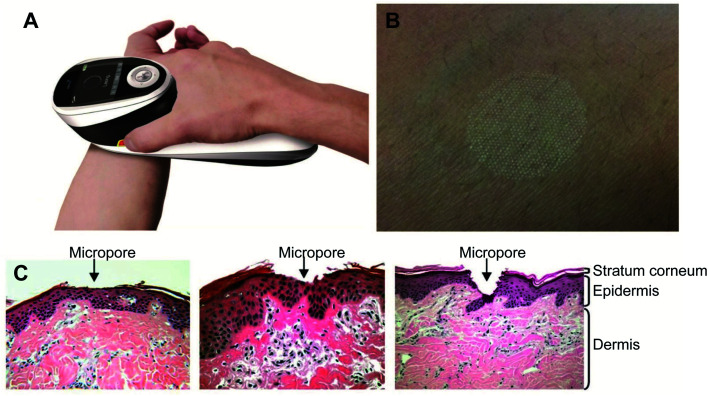
P.L.E.A.S.E.® technology. (A) The photograph of the hand-held device. (B) Formation of a micropore array in the skin surface using the device. (C) Hematoxylin and eosin staining of micropores created in porcine ear skin after laser microporation using the device at fluences of 4.53 J cm^−2^, 22.65 J cm^−2^ or 135.9 J cm^−2^ (from left to right). Reprinted with permission from ref. 99, Degruyter, Copyright 2013.^99^

Ramadan and his coworkers have synthesized HCuSNPs with an average 55 nm diameter. These NPs have an optical absorption band at around 1050 nm with 80% peak absorption intensity at 900 nm. They dispersed the NPs in Carbomer 940 hydrogel before applying them topically to the skin of mice. Propylene glycol (10%) gel was also added as a wetting agent to enhance the interaction between the skin and the gel. Discontinuous laser light from a Nd:YAG source (power density, 1.3 W cm^−2^) for a short time raised the skin temperature from 40–50 °C. Only the stratus corneum was damaged and there was no apparent harm to the viable epidermis. The pore depth was 11 ± 2 μm. The HCuSNPs, loaded with the hydrophilic drug fluorescein isothiocyanate (FITC) labeled dextran, were successfully up taken through the disrupted stratum corneum. Fluorescence microscopy and thermographic studies showed that heating of the epidermis was limited to a restricted area and there was an enhanced penetration of the FITC dextran. Similar results were obtained using a macromolecular drug – a human growth hormone. This procedure guarantees the effective transport of hydrophilic medications, vaccines, and proteins, which may not be amenable to intravenous or oral administration^98^ (Fig. 11).

### 3.2 Copper sulfide applications as sensors

#### 3.2.1 Glucose sensors

For the use of copper sulfide NPs in glucose sensors, a three-electrode system has been employed using a platinum wire as the auxiliary electrode, a standard calomel electrode as the reference, and a copper sulfide-modified electrode as the working electrode. Electrochemical experiments were performed in a cell containing a 7.2 pH phosphate buffer solution and the observation of a high anodic peak for the copper sulfide-modified electrode illustrated the electrocatalytic response towards glucose oxidation.

**Fig. 12:**
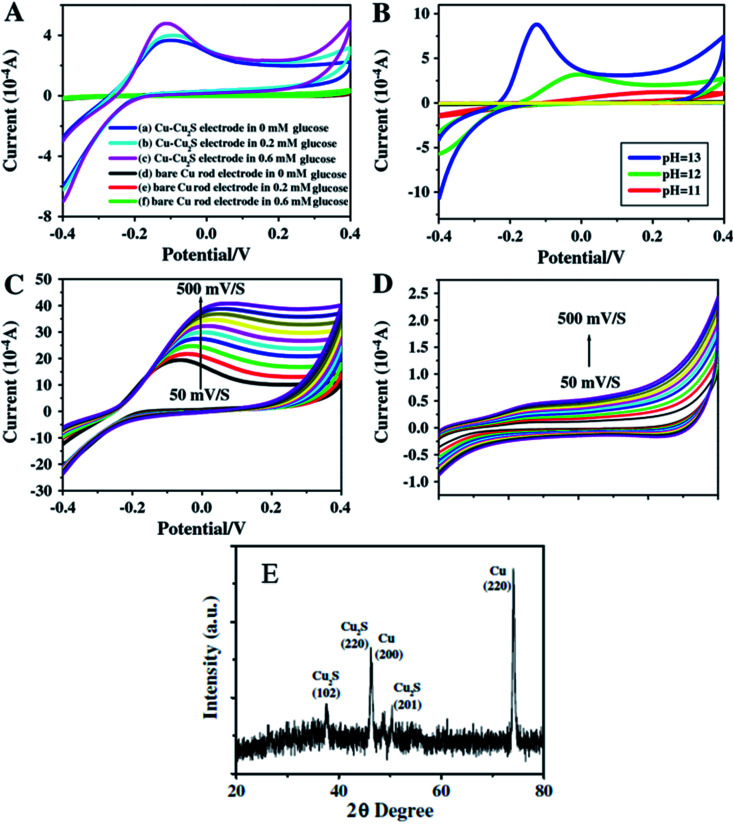
CV images for Cu–Cu_2_S nanocomposite electrodes (A) and bare Cu rod electrodes (B) in pH = 13 of NaOH and different concentration of glucose of various pH and the concentration of glucose is 0.4 mM; (C) Cu–Cu_2_S nanocomposite electrodes pH = 13 0.4 mM glucose at different scan rates (50–500 mV s^−1^); (D) bare Cu rod electrodes pH = 13 0.4 mM glucose at different scan rates (50–500 mV s^−1^); (E) XRD pattern of Cu_2_S. Reprinted with permission from ref. 100, Elsevier, Copyright 2012.^100^

Zhang and coworkers^100^ have prepared Cu–Cu_2_S nanocomposites and utilized them as working electrodes together with a calomel electrode (as reference) and a platinum electrode. The structural analysis was carried out *via* PXRD analysis. The presence of two peaks (200) and (220) were attributed to metallic copper while other peaks at (220), (102) and (201) were indexed to Cu_2_S {Fig. 12(E)}.

The electrochemical properties of Cu–Cu_2_S nanocomposites with respect to glucose oxidation were investigated by cyclic voltammetry and amperometric techniques for a glucose mixture containing 0.1 mM NaOH. A bare Cu electrode showed only a weak amperometric response towards different concentrations of glucose in a 0.1 M NaOH solution as compared to the much better results obtained with the Cu–Cu_2_S nanocomposites.

The current response increased with an increase in the glucose concentration indicating high catalytic activity of the electrode towards glucose oxidation. The anodic peak current increased linearly with scan-rate ranges from 50–500 mV s^−1^. The stability of the electrode was confirmed by adding interference materials (uric acid, ascorbic acid). These additives did not affect the glucose response at the Cu–Cu_2_S electrode and the detection limit was 0.1 μM (ref. 100) (Fig. 12).

**Fig. 13:**
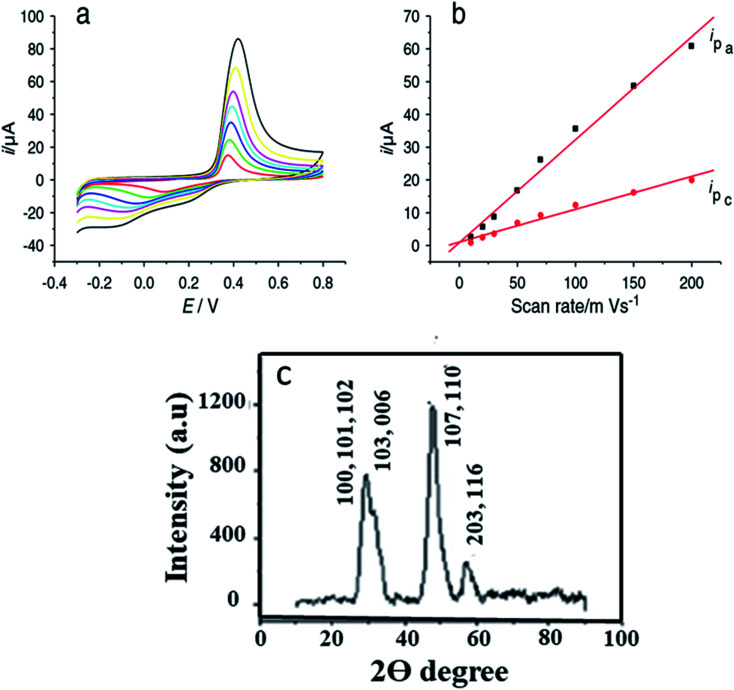
(a) Cyclic voltammograms of CuS/chitosan/GCE at 0.01, 0.02, 0.03, 0.05, 0.07, 0.1, 0.15 and 0.2 V s^−1^ in 0.1 M pH 7.2 phosphate buffer solution; (b) logarithmic plot between the peak current *I*_p_ (μA) and the scan rate (V s^−1^) for CuS/chitosan/GCE; (c) XRD pattern of CuS. Reprinted with permission from ref. 101, MDPI, Copyright 2021.^101^

Yang and coworkers^101^ have prepared copper sulfide NPs by a simple aqueous route. The XRD analysis showed the formation of diffraction peaks that were well indexed to hexagonal CuS with JCPDS card no. 78-0876. The non-crystalline nature of CuS was explained by the broadening of peaks {Fig. 13(c)}.

They modified the surface of Glassy Carbon Electrode and used GCE/chitosan/CuS as the working electrode for glucose oxidation in the presence of a 0.1 M phosphate buffer solution. CV measurements were recorded with continual 1 × 10^−4^ M glucose addition. The cathodic peak current (−0.1 V) and the anodic peak current (0.42 V) amplified linearly with an increase in glucose concentration from 1 × 10^−4^ M to 1 mM. Glucose was oxidized to gluconolactone by Cu(ii) directly. The Cu(i) produced experienced further two reactions^101^ (eqn (1)–(4) and Fig. 13).

1 2Cu(ii) + glucose → 2Cu(i) + gluconolactone + 2H^+^

2 2Cu(i) + O_2_ + 2H^+^ → 2Cu(ii) + H_2_O_2_

3 Cu(i) − e^−^ → Cu(ii)

4 H_2_O_2_ + 2e^−^ → 2OH^−^

**Fig. 14:**
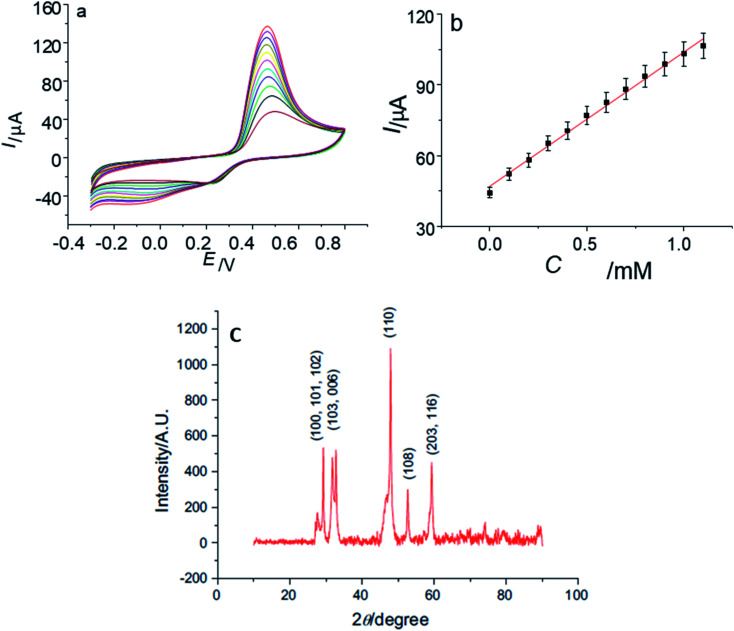
(a) CVs of CuS/CS/GCE in 0.10 M phosphate buffer solution (pH 7.2) containing 0, 0.1, 0.2, 0.3, 0.4, 0.5, 0.6, 0.7, 0.8, 0.9, 1.0, 1.1 mM glucose at 100 mV s^−1^; (b) the plot of the oxidation peak current with the concentration of glucose; (c) XRD data of synthesized CuS Nanoparticles. Reprinted with permission from ref. 102, Elsevier, Copyright 2013.^102^

In another study by Yang' group,^102^ flower-like copper sulfide NPs were fabricated together with chitosan on a GCE. The diffraction peaks in the PXRD pattern {Fig. 14(c)} explained the polycrystalline nature of synthesized material and were indexed to hexagonal CuS NPs. The absence of impurity peaks confirmed the purity of the sample.

The CV of the CuS/CS/GCE was recorded upon adding 100 μM glucose. The anodic peak at 0.46 V was improved with an increase in glucose concentration in the linear range of 1 × 10^−5^ M to 1 × 10^−2^ M. This range is important because the level of glucose in both normal and diabetic patients is typically 0.2 to 20 mM. No change in activity (current response) of the electrode was observed, even after storage for 30 days at room temperature. The synthesized electrode was also stable to interfering biomolecules^102^ (Fig. 14).

**Fig. 15:**
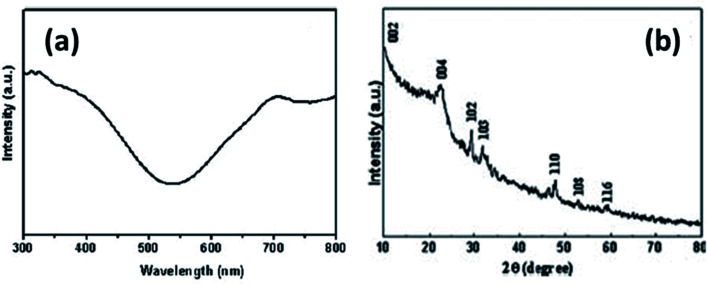
(a) UV-Vis and (b) XRD spectrum of CuS nanotubes. Reprinted with permission from ref. 103, Royal Society of Chemistry, Copyright 2011.^103^

In yet another study by Liu and coworkers, a copper sulfide nanotube-modified electrode was developed.^103^ The structural analysis was confirmed *via* PXRD that were well indexed to hexagonal CuS nanostructures with JCPDS no. 75-2234. In addition, the UV-Vis Spectroscopic analysis exhibited a broad shoulder peak at 325 nm that reached to its minimum at 540 nm with low intensity. Another absorbance peak at a longer wavelength appears which is due to free carrier intra-band absorbance {Fig. 15(a and b)}.

This nanoporous nanotube exhibited a greater electrocatalytic activity towards glucose oxidation. The transfer of electrons between the electrode and glucose was enhanced. An increase in glucose concentration amplified the current in the linear range of 0.5–7.5 mmol L^−1^ with a detection limit of 250 μM. The electrode was stable with high reproducibility.^103^

**Fig. 16:**
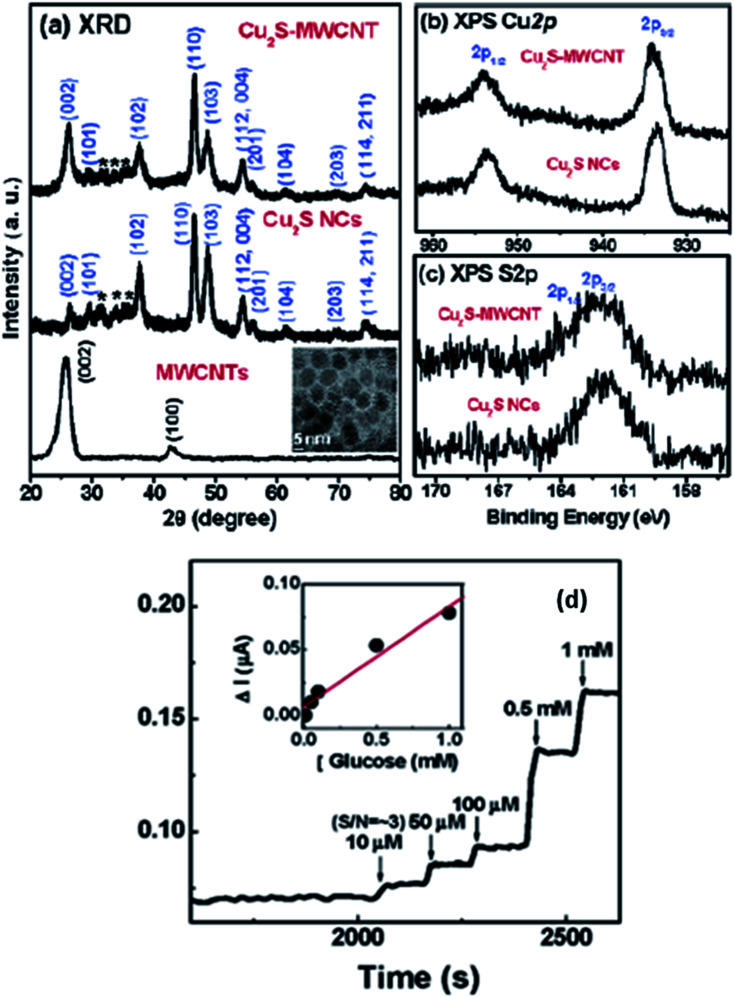
(a) XRD patterns for acid-functionalized MWCNTs, free Cu_2_S NCs, and Cu_2_S-MWCNT. The inset displays the TEM image of free Cu_2_S NCs. Fine-scanned XPS of (b) Cu 2p_1/2_ and 2p_3/2_, (c) S 2p_1/2_ and 2p_3/2_ of free Cu_2_S NCs and Cu_2_S-MWCNT and (d) CA response of Cu_2_S-MWCNT/GOx/Nafion electrodes upon the addition of glucose solution. The detection limit is 10 μM. The inset shows the linear response for glucose concentrations between 10 μM and 1 mM. Reprinted with permission from ref. 104, American Chemical Society, Copyright 2007.^104^

Lee *et al.* have managed to prepare Cu_2_S on the surface of multiwalled carbon nanotubes (MWCNT) and synthesized Cu_2_SMWCNT/GOx/Nafion-modified electrode.^104^ Structural analysis of synthesized material was carried out *via* XRD. Graphite showed the diffraction peaks at (002) and (100), whereas Cu_2_S nanoparticles resembled well with the hexagonal chalcocite phase (JCPDS 26-1116). The average size calculated *via* the Debye–Scherrer equation was found 10 nm. The weak intensity peaks observed between 30 and 40° marked with asterisk were due to the presence of surplus sulfur present on the Cu_2_S surface. The XPS analysis also explained the Cu_2_S formation on the surface of MWCNT. The XPS spectrum of Cu_2_S-MWCNT illustrates the presence of C, O, Cu, and S. Fig. 16(b) shows well-resolved peaks of Cu 2p_3/2_ and 2p_1/2_ peaks in addition to those of free Cu_2_S that became broader to some extent when the Cu_2_S are grown on the surface of MWCNTs. The unresolved peaks of S 2p_3/2_–2p_1/2_ of Cu_2_SMWCNT were also found broader than that of free Cu_2_S NCs {Fig. 16(c)} which can be attributed to the interaction of their electronic states with the graphite layers. The absence of obvious shoulder peaks in the region of higher energy explains the stability of the Cu_2_S surfaces.

This electrode is responsive to the successive addition of glucose. The inset in Fig. 16(d) displays a linear response with an increase in glucose concentration from 10 μM to 1 mM with the detection limit being 10 μM. This improved glucose biosensor showed a sensitivity of 75 nA mM^−1^. The detection limit of the Cu_2_S-MWCNT-based GC electrode is comparable to that for the Pt NC-SWCNT/Nafion GC electrode reported by the Lee group.^104^

#### 3.2.2 DNA sensors

A biosensor is a small device based on biological recognition properties which relies on the pairing of a biological recognizing element with a physical transducer to convert a biological signal to an electrical signal analogous.^105–110^ This technique has the advantage of no sample preparation with the ability to analyze on-site with low cost and rapid measurements.

Biosensor assembly mainly involves three parts receptors, transducers, and processors while sensing elements may include complete cells, nucleic acids, antibodies, or enzymes making a recognition layer which is then integrated with a transducer. Integration can be done *via* a number of techniques such as immobilization by adsorption, covalent binding, or cross-linking. Transducers work on many principles; which may be potentiometric,^111^ amperometric,^112^ thermal, piezoelectric,^113^ or optical.^114^ Basic analytical techniques require many steps with much labor, money, and time. Contrary to this biosensors are rapid and economical and can be easily used in remote areas where expensive instruments are out of reach.

Recently, nucleic acids have received great consideration in biosensors owing to their diverse chemical, physical and biological characteristics. These sensors employ oligonucleotides as a sensing element with a DNA/RNA fragment or a known base sequence. These biosensors either work as a receptor of a specific species or based on specific hybridization of complementary DNA/RNA.^115–117^ Nucleic acid-based sensors are more promising to obtain sequence-specific information in a much faster way in comparison to the traditional methods. They can be readily prepared and can be made more sensitive or specific in their combination with polymerase chain reactions (PCR).

DNA biosensors are highly imperative for the analysis of infectious diseases and gene mutations, that's why they are highly recommended in forensic and clinical laboratories. DNA biosensors play an important part in detecting the DNA sequence and various biosensors are available for particular DNA sequences.

**Fig. 17:**
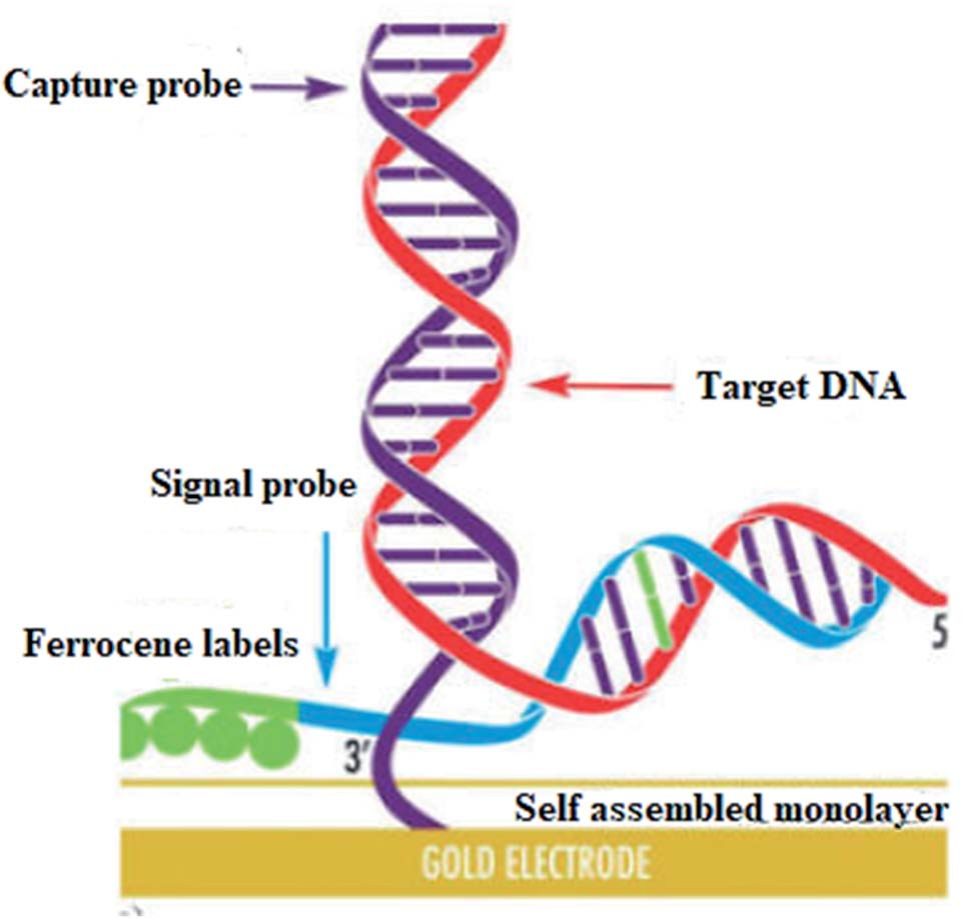
Self assembled monolayer. Reprinted with permission from ref. 118, Elsevier, Copyright 2017.^118^

DNA hybridization biosensors are based on the complementary DNA base pairing technique. In this method highly targeted single-stranded DNA segment with 20–40 base pairs is immobilized on the electrode surface in such a way that their stability and reactivity are retained (Fig. 17).

They are optimally oriented on the electrode surface to make their accessibility possible to the analyte. In hybridization, an electrical signal is produced on the binding of target DNA with the complementary strand of the capture/probe. Electrochemical indicators like ferrocenyl naphthalene diimide (FND) can be used to produce an electrical signal on binding with DNA duplexes. Alkaline phosphatase and horseradish peroxidase have commonly used enzyme labels that can efficiently measure the degree of hybridization. In addition, experimental conditions like ionic strength, temperature, and time need to be optimized to achieve high sensitivity and selectivity in hybridization.

Electrochemical biosensing has got considerable attention owing to its simplicity of analysis and microelectronic integration.^119^ The reduction in the size of the device along with its advanced technology makes them very efficient for DNA diagnosis. This technique is based on the measurement of current at the fixed potential. Here the nucleic acid probes are immobilized on the surface of the transducer.^118,120^

In contrast, classical chemiluminescence methods involve the luminescence of the enzyme (label) joined to probe DNA upon its hybridization with the target DNA but this method involves enzyme's poor stability and lower detection limit. Some silver and gold nanoparticles are also reported as bio labels in DNA sensors but their instabilities in the water remain a disadvantage. Contrary to this, Cu^2+^ is more effective as a bio label owing to its enhanced solubility in water and low cost.

**Fig. 18:**
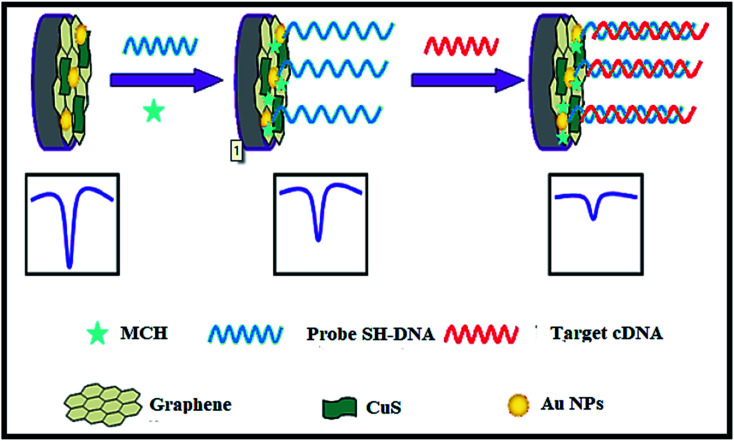
The electrochemical DNA biosensor consists of graphene, CuS, Au NPs, target complementary DNA, MCH and 6-mercapto-1-hexane. Reprinted with permission from ref. 121, Springer, Copyright 2014.^121^

CuS–graphene nanocomposites were synthesized as a DNA biosensor. The synthesized nanocomposite comprised of CuS NPs, gold NPs-conjugated ssDNA probe, graphene nanosheets, and MCH (Mean Cell Haemoglobin) (Fig. 18). The inserting of CuS in graphene nanosheets enhanced the solubility and dispensability of graphene nanosheets with improved CuS conductivity. The electrochemical response of [Fe(CN)_6_]^3−/4−^ decreased on hybridizing the target DNA strand with a DNA probe to form a double-stranded structure on the surface of the biosensor, thus leading to a low detection limit for the target DNA.

A biosensor for a short DNA sequence based on the chemiluminescence technique was reported. Luminol–H_2_O_2_–Cu^2+^ CuS nanotags were used on probe DNA to generate a chemiluminescence signal. This signal is produced as a result of the dissolution of Cu^2+^ on hybridizing target DNA with the probe DNA. The intensity of the signal was found to increase linearly with the target concentration (detection limit 5.5 × 10^−13^ M).^122^ In comparison to silver nanoparticles luminol–H_2_O_2_–Cu^2+^ was found to be less expensive, simple, fast, and easier to construct. Additionally, DNA modification with CuS requires much less time (13 h) than Ag NPs (116 h). The same group reported a new sensor with less detection limit and high sensitivity. In this sensor, the signal amplification ability of gold and Cu^2+^ was employed simultaneously. This combined system provided a low detection limit of 4.8 × 10^−15^ M of a target with good specificity.

Single-nucleotide polymorphisms (SNPs) mainly discuss point mutations that are responsible for genetic variations in human beings. A single alteration in the base can lead to various medical and physiological problems in the human body.^121^ Therefore correct analysis of single-nucleotide polymorphism is necessary for disease diagnosis, assessment, and evolutionary studies.^123^ In a study, copper sulfide-based nanostructures were synthesized to examine SNPs. This study involved modification of the gold surface with CuS NPs that are then conjugated with the complementary strands of the base. Cupric ions were found to be dissolved from CuS nanostructures on hybridization with mismatched base resulting in a chemiluminescence signal with the SNPs detection limit of 8.0 × 10^−17^ to 1.0 × 10^−14^ M.

## 4. Conclusions and future perspectives

Cu_*x*_S_*y*_ nanostructures are promising materials for both sensing and bioimaging applications. The use of Cu_*x*_S_*y*_ NPs is not limited to just cancer but they can also be used for the treatment of many other *in vitro* antibacterial diseases owing to their versatility and multifunctional properties.^124^ This review has focused mainly on the synthesis, characteristics, and applications of Cu_*x*_S_*y*_ nanostructures as biosensors, and their use in the analysis and treatment of cancer. Various synthetic methods have been described in this article to modify the surface of Cu_*x*_S_*y*_ NPs that can help them efficiently perform their activities and also facilitate their easy removal from the body. Numerous animal experiments have been undertaken to study the effect of Cu_*x*_S_*y*_ NPs in photoacoustic imaging, photothermal ablations, and drug delivery, but much more work needs to be done to develop Cu_*x*_S_*y*_ with more desirable properties.

The research on Cu_*x*_S_*y*_ nanostructures is, however incomplete yet, and many challenges need to be overcome. Cu_*x*_S_*y*_ NPs have a very low photothermal ratio and require the application of high-intensity lasers, which can cause severe damage to healthy tissues thus limiting the possible *in vivo* applications. The size, morphologies, and properties of the NPs can be tuned by making use of surface ligands that can improve the photothermal ratios. Much work is required to elucidate the pharmacokinetics and tolerance in mammals before these materials can be envisaged for clinical use.

More efforts are needed to further increase the transdermal drug delivery ability of copper sulfide NPs. This can be achieved by improving their surface chemistry and morphologies, and can also be modified by designing and coating hollow and porous nanospheres with polymer materials. The use of copper sulfide NPs to be used as efficient electrode material for sensing applications can be amended to higher level by refining their stoichiometries. Various composite materials can be combined with copper sulfide materials to enhance their electrical conductivity. The biomedical applications of copper sulfide NPs need inclusive investigation to scale-up laboratory studies to an advanced level. The design of robust and effective copper sulfide NPs based systems remains a foremost future challenge. This review exposed some of the imperative advances that need to be accomplished.

## Conflicts of interest

There is no conflict of interest.

## Supplementary Material
